# Study rationale and protocol: prospective randomized comparison of metal ion concentrations in the patient's plasma after implantation of coated and uncoated total knee prostheses

**DOI:** 10.1186/1471-2474-10-128

**Published:** 2009-10-14

**Authors:** Jörg Lützner, Gerd Dinnebier, Albrecht Hartmann, Klaus-Peter Günther, Stephan Kirschner

**Affiliations:** 1 Department of Orthopaedic Surgery, University Hospital Carl Gustav Carus, Medical Faculty of the Technical University of Dresden, Germany; 2 Institute of Clinical Chemistry and Laboratory Medicine, University Hospital Carl Gustav Carus, Medical Faculty of the Technical University of Dresden, Germany

## Abstract

**Background:**

Any metal placed in a biological environment undergoes corrosion. Thus, with their large metallic surfaces, TKA implants are particularly prone to corrosion with subsequent release of metal ions into the human body which may cause local and systemic toxic effects and hypersensitivity reactions, and increase cancer risk. To address this problem, a new 7-layer zirconium coating developed especially for cobalt-chrome orthopaedic implants was tested biomechanically and found to lower metal ion release.

The purpose of the proposed clinical trial is to compare the metal ion concentration in patients' plasma before and after implantation of a coated or uncoated TKA implant.

**Methods/Design:**

In this randomised controlled trial, 120 patients undergoing primary TKA will be recruited at the Department of Orthopaedic Surgery of the University Hospital in Dresden, Germany, and randomised to either the coated or uncoated prosthesis. Outcome assessments will be conducted preoperatively and at 3 months, 12 months and 5 years postoperatively. The primary clinical endpoint will be the chromium ion concentration in the patient's plasma after 1 and 5 years. Secondary outcomes include cobalt, molybdenum and nickel ion concentrations after 1 and 5 years, allergy testing for hypersensitivity against one of these metals, the Knee Society Score to assess clinical and physical function of the knee joint, the self-assessment Oxford Score and the Short Form 36 quality of live questionnaire.

**Discussion:**

The metal ion concentration in the patient's plasma has been shown to increase after TKA, its eventual adverse effects being widely debated. In the light of this discussion, ways to reduce metal ion release from orthopaedic implants should be studied in detail. The results of this investigation may lead to a new method to achieve this goal.

**Trials register:**

Clinicaltrials registry NCT00862511

## Background

Total knee arthroplasty (TKA) is a very successful treatment option for advanced osteoarthritis of the knee with about 146.000 operations performed in 2008 in Germany [1]. Although patients obviously benefit from joint replacement in terms of mobility and quality of life, implant-specific local and systemic adverse effects, due to corrosion and wear, still constitute a matter of concern [2-6]. Besides polyethylene wear which is one cause for aseptic loosening, metal ion release from metallic implants is also subject of debate. Every metal implant in a biological environment corrodes, depending on the exposed surface area and the composition of the alloy [7]. This process results in the release of metal ions.

It has been suggested that metal ion release may trigger local and systemic toxic effects [8] and hypersensitivity reactions [9-12]. Furthermore, in animal studies, cobalt and chromium ion exposure was found to have carcinogenic and mutagenic effects. Therefore, cancer risk may increase from exposure to the cobalt and chromium ions released by metal implants [13-15]. In contrast to these experimental data, epidemiologic studies did not find a greater risk of developing a malignancy after TKA [6,16,17]. However, increases in lymphoma, cancer of the endometriuma and prostate and skin melanoma were observed that could be related to TKA [16,17].

As they contain large metallic surfaces, TKA implants are particularly subject to corrosion inducing release of metal ions. Significantly higher metal ion concentrations were detected in patients after TKA compared to patients without metal implants [18]. Attempts have been made to reduce metal ion release and to improve the tribological properties of TKA implants by coating the chrome-cobalt body with various zirconium compositions, especially designed for patients with metal hypersensitivity. In in-vitro biomechnical tests, they showed superior tribological characteristics and thus less polyethylene wear particles [19-22]. However, in clinical tests, they presented early radiolucent lines [23] and achieved less favourable results in the Australian TKA registry [24].

To reduce ion release, a new multilayer coating system was developed consisting of a thin adhesive chrome layer, five alternating intermediate layers out of chrome nitride (CrN)-chrome carbonitride (CrCN) and a final shielding zirconium nitride (ZrN) layer.

The 7-layer coating system is applied on the CoCrMo knee implants through the chemical vapour deposition (CVD) method with a total thickness of about 3,5 to 6 μm. The gradiently applied CrN-CrCN layers allow to bridge the difference in hardness and tension between the softer material CoCrMo and the very hard ZrN shielding layer and thus ensure the system's mechanical integrity. The interfaces between the layers constitute an additional diffusion barrier against ions from the base material. In addition, the final ceramic ZrN layer seals the system and is highly biocompatible.

To evaluate its abrasive properties, it was tested in an in-vitro simulation according to ISO 14243-1:2002(E) through 5 million cycles with a frequency of 1 Hz.

Nickel and molybdenum ion release of the ZrN-coated implants into the wear test serum was reduced to the point that traces of these elements were found only within the range of detection levels while only minimal traces of cobalt and chrome were detected in the test serums, inferior by orders of magnitude to those of the uncoated implants out of CoCrMo [25].

Therefore, this surface coating could help to significantly reduce release of metal ions in TKA patients.

The aim of this study is to compare this novel surface coating with the standard CrCoMb-alloy in terms of metal ion levels in patients' plasma, metal hypersensitivity and clinical and radiological results.

### Study Hypothesis

The study's hypothesis is that metal ion concentrations in the patient's plasma will be significantly lower in the group receiving the ZrN-coated implant than in the other group.

## Methods/Design

This study is a single-centre randomised controlled trial that will be conducted at the Medical Faculty of the Technical University of Dresden. The study protocol was approved by the local independent Ethics Committee in February 2009 and registered in the US National Institutes of Health's database  registry under NCT00862511.

The trial is performed according to the guidelines for Good Clinical Practice (GCP), as applicable.

### Inclusion and Exclusion Criteria

Potential participants will be screened and recruited at the orthopaedic pre-admission clinic.

**Figure 1:**
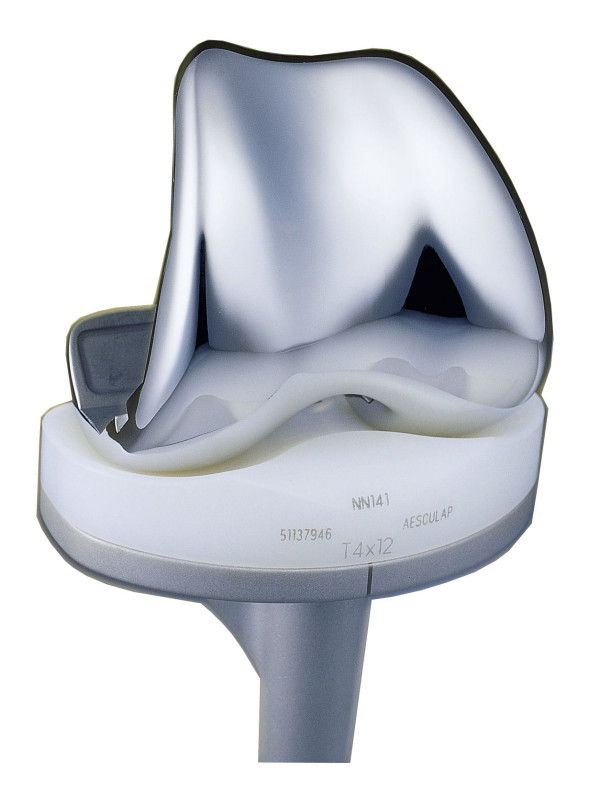
**View of the Columbus knee system (Aesculap, Tuttlingen, Germany)**.

**Figure 2:**
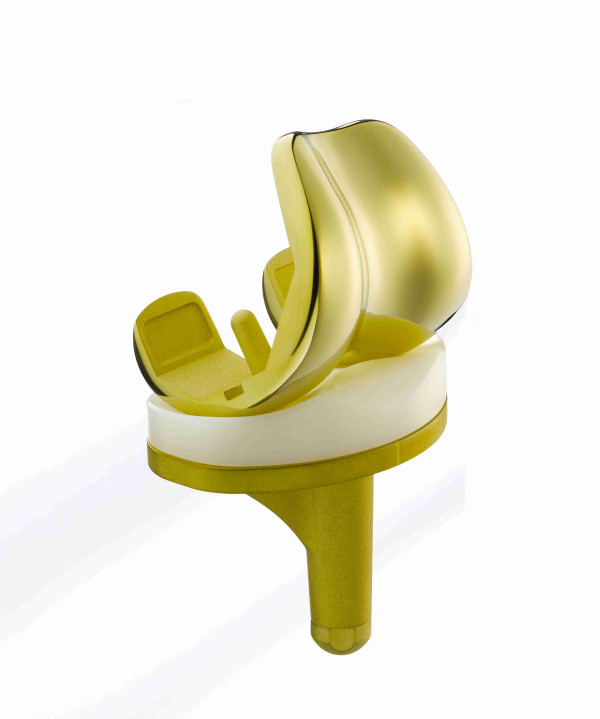
**View of the the ZrN-coated Columbus AS knee system (Aesculap, Tuttlingen, Germany)**.

Inclusion criteria are: (i) indication for TKA in cases of primary or secondary osteoarthritis, (ii) written informed consent. Exclusion criteria include (i) metal implants or endoprostheses, (ii) hypersensitivity against metals or bone cement particles, (iii) any form of malignancies, (iiii) renal insufficiency, (iiiii) other severe illnesses that would impair participation in this study. All eligible patients will be asked whether they are willing to participate in the trial and, if so, required to provide a written informed consent. They will be informed about the purpose of the trial, the operative procedures as well as their options and risks. They will be randomised to either a coated or uncoated TKA implant (Columbus, Aesculap, Tuttlingen, Germany, see figure 1 & 2) using a randomisation list.

### Operative Procedure

In all cases, the coated or uncoated model of a cemented unconstrained total knee prosthesis with a fixed polyethylene insert is implanted. Both implants are CE-certified.

All operations are performed with a tourniquet after a single dose of antibiotics (1.5 g Cefuroxim), using a medial parapatellar approach with a femur-first preparation.

Femoral alignment in the frontal plane is is directed at an angle of 90° to the mechanical axis in the frontal plane. For sagittal alignment, it is placed parallel to the ventral femoral cortex and, for rotational alignment, parallel to the transepicondylar axis. Tibial alignment is directed at an angle of 90° to the mechanical axis in the frontal plane, with a 5° posterior slope in the sagittal plane and at the medial third of the tibial tubercle for rotational alignment. Postoperatively full weight-bearing is allowed, and mobilisation starts from the first postoperative day.

### Clinical Endpoints

The primary endpoint of this investigation is the difference in chromium ion concentration in the patient's plasma after 1 and 5 years. Secondary endpoints are the differences in cobalt, molybdenum and nickel ion concentration after 1 and 5 years and a possible hypersensitivity against one of these metals after 1 year postoperatively.

The metal ion concentrations are to be analysed for a potential correlation to biomechanical factors (BMI, mechanical axis), activity (UCLA), function (KSS) and quality of live (Oxford Score, SF 36).

The patients will be clinically examined preoperatively and 3 months, 1 year and 5 years after surgery. They will be asked to complete the Knee Society score, the Oxford Knee score, the Short Form 36 and the UCLA activity score questionnaires. They will be tested for hypersensitivity against metal ions preoperatively and one year postoperatively. They will be contacted by phone 2, 3 and 4 years postoperatively and asked to complete the Oxford Knee score questionnaire. Adverse and serious adverse events will be evaluated according to Good Clinical Practice (see table 1 for details).

**Table 1:** Summary of the study parameters

	**Preop**.	**Discharge** **7 - 10 days** **postop**.	**Follow-up** **3** **months**	**Follow-up** **12 months**	**Follow-up** **2 years**	**Follow-up** **3 years**	**Follow-up** **4 years**	**Follow-up** **5 years**
Clinical examination	**X**		**X**	**X**				**X**
Contact by phone					**X**	**X**	**X**	
Metal ion concentrations in plasma	**X**			**X**				**X**
Testing for hypersensitivity	**X**							**X**
Knee Society score	**X**		**X**	**X**				**X**
Oxford score	**X**		**X**	**X**	**X**	**X**	**X**	**X**
UCLA activity score	**X**		**X**	**X**				**X**
SF 36	**X**		**X**	**X**				**X**
AE/SAE		**X**	**X**	**X**	**X**	**X**	**X**	**X**

### Metal Ion Analysis

Blood samples are collected in 7,5 ml S-Monovette^® ^tubes (for trace metal analysis, Sarstedt AG, Germany) using a specific steel needle for trace metal analysis (Sarstedt AG, Germany). Within one hour, plasma is separated by centrifugation at 2000 g for 10 min. Samples are stored at -20°C before being analysed for chromium, cobalt, molybdenum and nickel content using a graphite furnace atomic absorption spectrometer Z-8270 with Polarisation-Zeeman-Absorption (Hitachi Ltd., Japan) [26,27]. Calibration is performed by the standard addition method using 0.00 μg/l, 5.00 μg/l and 10.00 μg/l as calibration points in triplicate for each element. The samples are diluted 1:2 in buffer (1% HNO_3 _[Merck AG, Germany], 0.2% Triton X-100 [SIGMA-Aldrich Chemie GmbH, Germany], 0.2% Antifoam B [SIGMA-Aldrich Chemie GmbH, Germany]; Cr and Co: additional 0.8% Pd-matrix-modifier [Merck AG, Germany], 0.3% Mg-matrix-modifier [Merck AG, Germany]). The accuracy and precision of the method is validated to < 10% using the control materials Seronorm™ Trace Elements Serum (SERO AS, Norway). The detection limit of the method is estimated at 0.5 μg/l for each element (mean +3sd from buffer). The Dixon test was used to eliminate aberrant values. All probes having ion levels below the detection levels are adjusted to <0.25 μg/l.

### Sample Size Considerations

To estimate the number of cases, own data from a cross-sectional study of patients with a CrCoMb alloy TKA was used [18]. The chromium ion concentration in the patient's plasma 5 to 7 years postoperatively was in average 0.92 μg/l (interquartile range 0.73 - 1.32 μg/l). For the difference between an uncoated and a ZrN-coated prosthesis, the data of a biomechanical study [25] was used. This in-vitro test demonstrated a 40 times higher chromium ion release in the uncoated implant. 3 coated and 3 uncoated implants were tested and the serum after 1 million loading cycles was taken for the measurement of metal ion concentration. The chromium ion concentrations was 201 μg/l for the uncoated implant and 4.5 μg/l for the coated implant.

Taking into account that a normal distribution of data can not be assumed, the relatively high age of the patients and an expected drop-out rate of 25%, the necessary number of cases was estimated at 60 patients per group when a significance level of 5% and a test strength of 80% are required.

### Data Acquisition and Management

Patients will be interviewed by a local study nurse pre-operatively and at specified time intervals postoperatively (see table 1 for details). In addition to the written interview, the Knee Society score, the Oxford Knee score, the Short Form 36 and the UCLA activity score will be recorded.

Data will be entered in prepared CRFs and then transferred to a SPSS database and subjected to range and plausibility checks. Data reported on the CRF derived from source documents (e.g. operative report) should be consistent with the source documents or the discrepancies should be explained.

The clinical database, including all information until 5 years postoperatively, will be closed after the 5-year follow-up visit of the last patient enrolled into the trial. There will be an interim analysis after the 1-year follow-up visit of the last enrolled patient which includes the evaluation of the primary endpoint and the secondary endpoints.

### Current Status and Planning

The study protocol was completed in December 2008. Preparation of all study-related material was finalised until February 2009. After approval from the local Ethics Committee on 26 February 2009, the first patient was recruited on 27 April 2009 and more patients are currently being enrolled at the centre. Assuming an enrolment of 6 to 8 patients per month, recruitment is expected to be completed at the end of 2010.

## Discussion

An "ideal" knee prosthesis would prevent release of any metal ions into the patient's blood and thus protect him/her from any local or systemic toxic effects, cancer risk or activation of the immune system possibly resulting in hypersensitivity against components of the prosthesis. Although risks of such adverse effects have not been yet conclusively proven, they cannot be excluded. Therefore, reduction of metal ion release through coating of an implant may be an advantage if disadvantages in terms of premature loosening can be avoided. This underscores the need to test these new implants in clinical trials.

## Conclusion

The present trial is a single-centre, prospective randomised trial to evaluate metal ion release of a novel zirconium-coated total knee prosthesis compared to an uncoated implant of the same geometry. It aims to find out whether this coated implant can reduce metal ion release and how both implants compare with respect to adverse events, functional results and quality of live.

## Competing interests

The authors declare that they have no competing interests, although this study is financially supported by Aesculap AG, Germany including the article processing-charge.

## Authors' contributions

JL, GD, AH, KPG and SK designed the trial together. JL wrote the manuscript with contributions from GD, AH, KPG and SK. GD is performing the metal ion analysis. SK is involved as statistician. JL, AH and SK are participating actively in the recruitment of the patients. All authors have read and approved this manuscript.

## Pre-publication history

The pre-publication history for this paper can be accessed here:


